# Common and distinct lateralised patterns of neural coupling during focused attention, open monitoring and loving kindness meditation

**DOI:** 10.1038/s41598-020-64324-6

**Published:** 2020-05-04

**Authors:** Juliana Yordanova, Vasil Kolev, Federica Mauro, Valentina Nicolardi, Luca Simione, Lucia Calabrese, Peter Malinowski, Antonino Raffone

**Affiliations:** 1 Institute of Neurobiology, Bulgarian Academy of Sciences, Sofia, Bulgaria; 2 Department of Psychology, Sapienza University of Rome, Rome, Italy; 3 Social and Cognitive Neurosciences Laboratory, IRCCS, Santa Lucia Foundation, Rome, Italy; 4 Institute of Cognitive Sciences and Technologies, CNR, Rome, Italy; 5 School of Psychology, Research Centre for Brain and Behaviour, Liverpool John Moores University (LJMU), Liverpool, UK; 6 School of Buddhist Studies, Philosophy and Comparative Religions, Nalanda University, Rajgir, India

**Keywords:** Cognitive control, Consciousness

## Abstract

Meditation has been integrated into different therapeutic interventions. To inform the evidence-based selection of specific meditation types it is crucial to understand the neural processes associated with different meditation practices. Here we explore commonalities and differences in electroencephalographic oscillatory spatial synchronisation patterns across three important meditation types. Highly experienced meditators engaged in focused attention, open monitoring, and loving kindness meditation. Improving on previous research, our approach avoids comparisons between groups that limited previous findings, while ensuring that the meditation states are reliably established. Employing a novel measure of neural coupling – the imaginary part of EEG coherence – the study revealed that all meditation conditions displayed a common connectivity pattern that is characterised by increased connectivity of (a) broadly distributed delta networks, (b) left-hemispheric theta networks with a local integrating posterior focus, and (c) right-hemispheric alpha networks, with a local integrating parieto-occipital focus. Furthermore, each meditation state also expressed specific synchronisation patterns differentially recruiting left- or right-lateralised beta networks. These observations provide evidence that in addition to global patterns, frequency-specific inter-hemispheric asymmetry is one major feature of meditation, and that mental processes specific to each meditation type are also supported by lateralised networks from fast-frequency bands.

## Introduction

Interest in meditation is burgeoning. Meditation and mindfulness exercises have been integrated into therapeutic interventions that target clinical conditions, including stress, chronic pain, depression and anxiety^1–5^. Meditation-based programmes are also offered in non-clinical contexts, for example, workplaces, education, sports, and criminal justice systems^6,7^. While the majority of these programmes aim to enhance mindfulness skills, a growing number also foster compassion, including self-compassion.

Typically, these programmes include a range of different meditation exercises. Although the reasons for including specific exercises in a programme often remain opaque, it is fair to assume that such choices were guided by the experience and preferences of the developers, rather than by scientific considerations. Arguably, in the early days of these intervention programmes, this approach was unavoidable because robust evidence about specific effects of distinct meditation types was unavailable. Furthermore, early meditation researchers sought insights about meditation practice in general, and in consequence did not offer a sufficiently nuanced picture of different meditation types. Meanwhile it has been acknowledged that a more fine-grained approach is needed. To put meditation and mindfulness interventions on more solid footing and encourage their evidence-based formulation and refinement, it is crucial to advance the understanding how different types of meditation work^8–12^.

Besides considering meditations for their therapeutic effects, scientists are also interested in studying highly experienced meditators – meditation virtuosos – who have honed their mental skills over many years and are able to maintain different meditative states at will^13–16^. Investigating neural activity of these virtuosos during different meditation states, offers a unique opportunity to understand what changes can be expected when people practice different types of meditation.

Toward this end, the present study investigated the neural states associated with three prominent and distinct meditation types, often referred to as *Focused Attention Meditation* (FAM), *Open Monitoring Meditation* (OMM) and *Loving Kindness Meditation* (LKM)^17–21^. While FAM and OMM are integral components of standard mindfulness-based programmes, such as mindfulness-based stress reduction (MBSR)^22,23^, LKM is included in programmes such as Compassion Cultivation Training (CCT)^24,25^ or Cognitive-Based Compassion Training to Children (CBCT)^26,27^. The distinction between FAM and OMM was brought to prominence by Lutz *et al*.^19^, who drew parallels between Buddhist meditation traditions and secular interventions, such as MBSR. Subsequently, Vago and Silbersweig^21^ and Dahl *et al*.^18^ broadened this conceptual framework by including LKM practices, also referred to as “Ethical Enhancement Practices”^21^ or as “constructive meditations” that nurture pro-social qualities^18^.

### Focused Attention Meditation (FAM)

FAM entails voluntary focusing attention on a chosen object in a sustained fashion. An important purpose of this meditation type is to enhance the cognitive skill of sustaining attention and to reduce emotional reactivity^19,28^. In principle, any object of the five senses as well as mental objects such as arising thoughts, feelings or even imagined visual objects can serve as reference point. As such, the exercise is not about an object per se, but about the ability to sustain focused attention. Within Buddhist contexts, the meditation type labelled here as FAM is often referred to by the Pali term *Śamatha*, typically translated as *Calm Abiding* meditation. Early stages of developing FAM or *Śamatha* are usually effortful, requiring meta-cognitive regulatory skills, such as recognising when the focus of attention is lost and the mind is distracted, disengaging attention from the source of distraction, and (re)orienting attention to the intended object^19,29–31^. Advanced practitioners are thought to require little – if any – effort to maintain focus, thus trivial regulatory skills will rarely need to be invoked^19,32,33^.

### Open monitoring meditation (OMM)

While FAM maintains a specific object as reference point, during OMM attention is not explicitly object-focused. It rather involves the monitoring of the contents of experience and of mental processes in the present moment. With practice, the ability to observe objects that arise in awareness without *selectively* attending to them becomes increasingly effortless. Secular mindfulness-based approaches see this non-engaged monitoring of mental patterns and habits, such as emotional reactions, judgements, and related dispositions as prerequisite for a transformation that leads to increased cognitive and emotional flexibilities^17,19,30,34,35^. Within Buddhist traditions, OMM might be termed “bare attention” or “choiceless awareness”. It is seen as a first step into the practice referred to as *Vipassanā*, translated as *Penetrating Seeing* or *Deep Insight Meditation*: Firmly established, OMM becomes a platform for exploring existential insights regarding the nature of experience and of the self^36,37^. Such insights are not the focus of this study. Nevertheless, it is worth noting that although the balanced monitoring of present moment experiences is common to secular and Buddhist OMM practice^31^, it serves different purposes depending on the practice contexts.

### The interrelation of FAM and OMM

Although FAM and OMM are commonly presented as two types of meditation, OMM requires some degree of mental stability and calmness. In both secular and Buddhist practices this is achieved by moving to OMM after initially practicing FAM. As FAM becomes less effortful, the explicit attentional reference point is phased out, while OMM is successively introduced. With other words, while the selective focus is removed, the monitoring function is increased. Rather than presenting a strict dichotomy, FAM and OMM can thus be seen as two meditations that regulate attention and meta-cognition with different apertures: FAM with a narrow attentional aperture and OMM with the widest possible aperture^18,31,38^.

### Loving kindness meditation (LKM)

LKM is a further type of Buddhist meditation that inspired secular applications^5,39–41^. Dahl *et al*.^18^ characterise LKM as a *constructive* meditation, which trains cognitive and affective mental states that implicate enhanced prosocial and empathetic feelings, attitudes and intentions. In particular, LKM focuses awareness upon a mental state of acceptance of self and others, and the intention (motivation) toward wellbeing and happiness of self and all others, with equanimity. LKM shares attributes with both FAM and OMM, with particular reference to openness and acceptance (equanimity), a core facet of mindfulness. Finally, LKM is often practiced in synergy with FAM and OMM for the reduction of unwholesome or unhealthy mental states and dispositions (e.g. anger, hatred), and for the development of wholesome or healthy mental states and dispositions (e.g., empathy, altruism, generosity, love, wisdom)^5,39,41^.

As outlined above, despite their different orientations, these three meditations appear to share common psychological processes, such as enhanced attentional stability and meta-cognition^31,42,43^. It is, however, not well understood (1) whether they cultivate a generic mental state that underlies meditation-specific cognitive and affective processes, (2) whether – despite apparent commonalities – these meditation states are profoundly different, or (3) whether they share some commonalities while also having distinct features. As highlighted in recent critical appraisals^11^, such questions need to be answered for utilising specific meditation types in a targeted way.

We were therefore interested in characterising three meditation types (FAM, OMM, and LKM) by identifying patterns of neural activity that are common to all three meditations and distinguishing these patterns from those that are specific to each type. Because FAM, OMM and LKM are very common meditation types – in secular and Buddhist contexts alike – investigating them promises to yield insights of broad relevance.

In addition, we aimed to use a neuroscientific approach that overcomes several methodological limitations of previous research and allows us to identify the dynamic coupling of brain networks during different meditation states: By recording brain activity during meditation with electroencephalography (EEG), meditators could stay in their accustomed meditation environment and assume their familiar meditation posture, sitting upright, rather than – for instance – laying in a confined supine position in a noisy fMRI scanner. By employing a refined measure of neural coupling – the imaginary part of coherency (*ICoh*) – we avoid two methodological problems that can lead to overestimation of neural coupling, namely the influence of volume conduction and the contribution of common sub-cortical sources^44^, as has been successfully used in other areas (e.g.^45–48^). Finally, by involving participants who are trained in all three meditation types, we reduced the influence of interindividual differences that could affect the comparison of meditation types, if they are carried out by different groups of participants.

### Brain networks of meditation

The evidence so far: Several recent reviews and meta-analyses summarise the evidence which brain structures and networks may be involved in FAM/OMM and LKM. In a meta-analysis of 78 functional neuroimaging studies (fMRI and PET), Fox *et al*.^49^ identified several brain areas, including insula, posterior dorsolateral prefrontal cortex (DLPFC), anterior cingulate cortex (ACC), and frontopolar cortex that were activated consistently across meditation types, when compared to control participants. The observation that several brain areas appear to be activated during all three meditation states, suggests common neuroactivation patterns. This interpretation is also supported by structural neuroimaging data. In another meta-analysis by Fox *et al*.^50^, several brain structures were different in experienced meditators of varied traditions when compared to participants with little or no meditation experience. These differences pertained to areas involved in body awareness (sensory cortices and insula), meta-awareness (frontopolar cortex), attention and emotion regulation (anterior and mid cingulate). Interestingly, also brain structures involved in intra- and interhemispheric communication (superior longitudinal fasciculus, corpus callosum) differed between meditators and controls, suggesting changes in connectivity of brain networks. However, in terms of specificity, the functional neuroimaging data are limited, because neither PET nor fMRI has the required temporal resolution for detecting more dynamic aspects of neural coupling. In addition, results from structural imaging do not differentiate between meditation types, because most – if not all – meditators will have engaged in several types of meditation.

To scrutinise more dynamic aspects, different meditation types have also been investigated using oscillatory neuroelectric brain signals of EEG and magnetoencephalographic (MEG) recordings. Recent reviews by Lomas *et al*.^51^ and Lee *et al*.^52^ summarise common and meditation-specific features of oscillatory activity, with a particular focus on activity in five well-established frequency bands: delta (1–4 Hz), theta (4–8 Hz), alpha (8–13 Hz), beta (13–30 Hz), and gamma (30–100 Hz). Although currently the evidence is sometimes ambiguous and tends to lack clear systematisation^52^, there appears to be some consistency across meditation types. Increased theta amplitudes have been associated with mindfulness meditation^51^, a feature confirmed by Lee *et al*.^52^ also for FAM, OMM, and LKM. On the other hand, evidence for other frequency bands and in particular for the alpha range, is more contradictory. For mindfulness meditation, Lomas *et al*.^51^ report that the majority of studies found an increase in alpha activity compared to a rest condition, whereas the review by Lee *et al*.^52^ found that frontal alpha was higher in OMM practitioners than in non-meditators and FAM-meditators. Similarly, Amihai and Kozhevnikov^53^ demonstrated that changes in alpha power might depend on the type of meditation: calming meditations in the Theravada Buddhist tradition reduced alpha power, whereas for activating meditations (in the Vajrayana Buddhist tradition) no difference to rest was detected. Thus, common and distinct patterns of oscillating activity in various frequency bands and for different meditation styles are currently not reliably established. One reason for the observed inconsistencies might be the heterogeneity of approaches, because most studies investigated only one meditation type and compared meditators against novice meditators, thus introducing potential interindividual differences. In addition, the focus on spectral power within different frequency bands offers only limited information about the involved networks.

Nevertheless, it is fair to say that functional and structural neuroimaging studies indicate that meditation states and traits may be associated with functional and structural neuroplasticity of major brain areas and networks, in particular attention networks, the central executive network, the salience network, and the default mode network^20,30,32,38,54^. According to several theoretical accounts, meditation may not only train process-specific brain networks, but also regulate integrated brain states, in interplay with autonomic nervous system states^10,55^. Moreover, given that large-scale integrative mechanisms in the brain also appear to play a crucial role for attention and conscious experience more generally^56–61^ it is plausible to expect that they are a significant aspect of meditation states^43,62^. Importantly, the functioning of large-scale networks is reflected in patterns of frequency-specific synchronisation of neuroelectric signals across brain regions^63^. However, as Lee et al.’s review showed, currently the knowledge of meditation-specific interregional coherence and connectivity patterns is limited^52^.

Against this backdrop, we aimed to improve the understanding of three common meditation types, thus, laying the groundwork for ‘prescribing’ meditation in a more targeted way than current evidence allows. Toward this end, we employed a within participant design that explored similarities and differences in EEG oscillatory synchronisation patterns during FAM, OMM, and LKM in meditation virtuosos from the Theravada Buddhist tradition. Involving highly experienced meditators, who regularly practice these meditations, allowed us to distinguish reliably between the three types of meditation.

The participants were 22 healthy volunteers (mean age 44.2 years, age range 26-70 years, 4 females), with an estimated mean lifetime meditation practice of 19,357 hours (range 900-50,600 hours). EEG was recorded from 64 electrodes with eyes closed while the virtuoso meditators maintained states of REST and FAM, OMM or LKM meditations. To explore patterns of spatial EEG synchronisation we analysed the imaginary part of coherence (*ICoh*) after spatially enhancing the EEG signals by applying current source density (CSD)^64^. We computed *ICoh* for all combinations of electrodes for each condition and investigated the absolute value of *ICoh*^65^ by applying several complementary approaches.

## Results

### Frequency specificity of connectivity in meditation conditions

**Figure 1:**
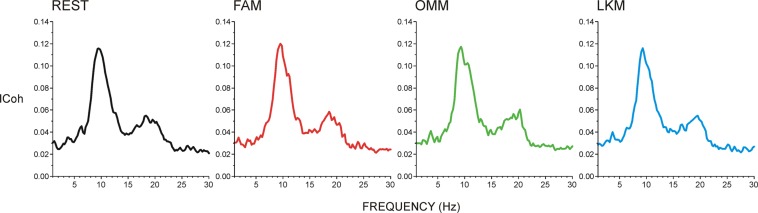
Grand average of the imaginary part of coherence (*ICoh*) as a function of frequency pooled for all investigated electrode combinations during resting state (REST), focused attention meditation (FAM), open monitoring meditation (OMM), and loving kindness meditation (LKM).

To identify the frequency specificity of networks during meditation in experienced meditators, frequency plots of *ICoh* were computed. Figure 1 presents *ICoh* grand averages of all electrode pairs in REST and three meditation conditions – FAM, OMM and LKM. It is demonstrated that network connectivity in all conditions is supported most prominently by networks in alpha (9–15 Hz) and beta (15–24 Hz) frequency bands. However, inspection of averages from individual participants revealed separable peaks in the delta and theta bands, as well as distinct peaks in the slow and fast alpha bands (also implied by grand-averages). Measurable parameters were therefore mean *ICoh* values for the following frequency ranges: delta (1.7-3.9 Hz), theta (4.1-7.1 Hz), slow alpha/alpha-1 (8.8-12 Hz), fast alpha/alpha-2 (12.2-14.9 Hz), and beta (15-20 Hz). Due to the lack of distinct peaks above 30 Hz in individual and grand average frequency plots of *ICoh*, gamma band was not evaluated.

### Condition-specific reorganisation of connectivity strength during meditation

The reorganisation of networks during meditation states was evaluated by comparing the number and localisation of strong and weak connections in each meditation condition (FAM, OMM and LKM) with those during REST.

**Figure 2:**
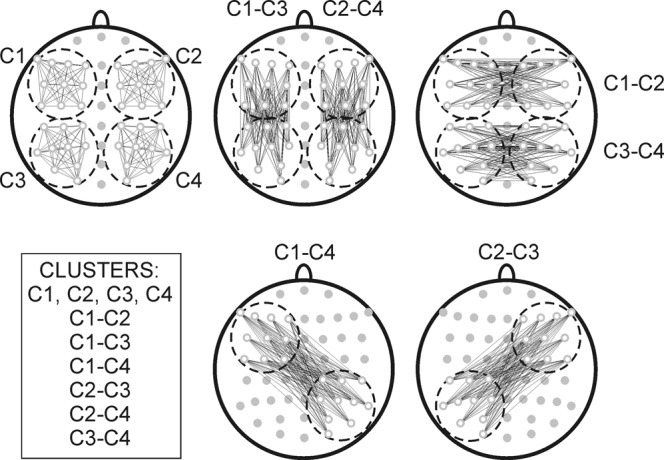
Schematic presentations of electrodes (in grey) and clusters used for analyses marked as C1, C2, C3, C4, C1-C2, C3-C4, C1-C3, C2-C4, C1-C4, and C2-C3. Electrodes in C1: F7, F5, F3, F1, FC5, FC3, FC1, C5, C3, C1; Electrodes in C2: F8, F6, F4, F2, FC6, FC4, FC2, C6, C4, C2; Electrodes in C3: CP5, CP3, CP1, P7, P5, P3, P1, PO7, PO5, O1; Electrodes in C4: CP6, CP4, CP2, P8, P6, P4, P2, PO8, PO6, O2. Inter- and intra-hemispheric connectivity is reflected by cluster-based measures of *ICoh* (mean of all electrode pairs between C1 and C2 for C1-C2, mean between all electrode pairs between C1 and C3 for C1-C3, etc.).

As illustrated schematically in Fig. 2, analyses were based on 10 regional clusters of electrode pairs. The aim of clustering was to evaluate connectivity within the left anterior (cluster C1), right anterior (cluster C2), left posterior (cluster C3) and right posterior (cluster C4) cortical areas, as well as intra-hemispheric antero-posterior connections in the left (cluster C1-C3) and the right hemisphere (cluster C2-C4), and inter-hemispheric connections between the anterior and posterior regions of two hemispheres (clusters C1-C2, C3-C4, C2-C4, C2-C3). The electrodes and electrode pairs included in each of the ten clusters are detailed in Fig. 2. The choice of these clusters did not target specific functional networks^62,63,66^. Rather, cluster creation considered the possible differential roles of anterior (frontal) and posterior (parieto-occipital) cortices in conscious regulation of information flow^59,67–69^, a possible lateralised involvement of the left hemisphere in meditation^20,49^, a right-hemisphere dominance of attentional networks controlling arousal and distraction^70–72^, as well as the established role for inter-hemispheric interactions in conscious perception^73–75^.

For each of the ten clusters, *ICoh* values from all pairs that belonged to a cluster were included in separate data sets, combining measures from REST and FAM, REST and OMM, and REST and LKM conditions. In this way, 30 separate data sets were created for each participant for each of the five frequency bands (delta, theta, slow alpha, fast alpha and beta, see above). For each data set (combining REST with one of the meditation states FAM, OMM, or LKM), the mean value and standard deviation (SD) were computed for each participant, and the number of pairs above and below the mean value plus SD (number of strong and weak connections, respectively) was determined for each state in each data set (REST vs. FAM, REST vs. OMM, REST vs. LKM). The distribution of strong and weak connections in REST vs. the corresponding meditation condition was evaluated using chi-square statistics to reflect if there is a prevalence of strong/weak connections in any of the 10 clusters during any meditation condition as compared to REST. Before being subjected to chi-square tests, individual numbers were summed up separately for strong and weak connections. The statistical significance of meditation-specific connectivity re-distribution was controlled using Bonferroni correction, where *p* was considered as significant if smaller than 3.10^−4^ following the correction *p*/*n* = 0.05/150, where n is the number of observations = 10 clusters × 5 frequency bands × 3 data sets.

**Figure 3:**
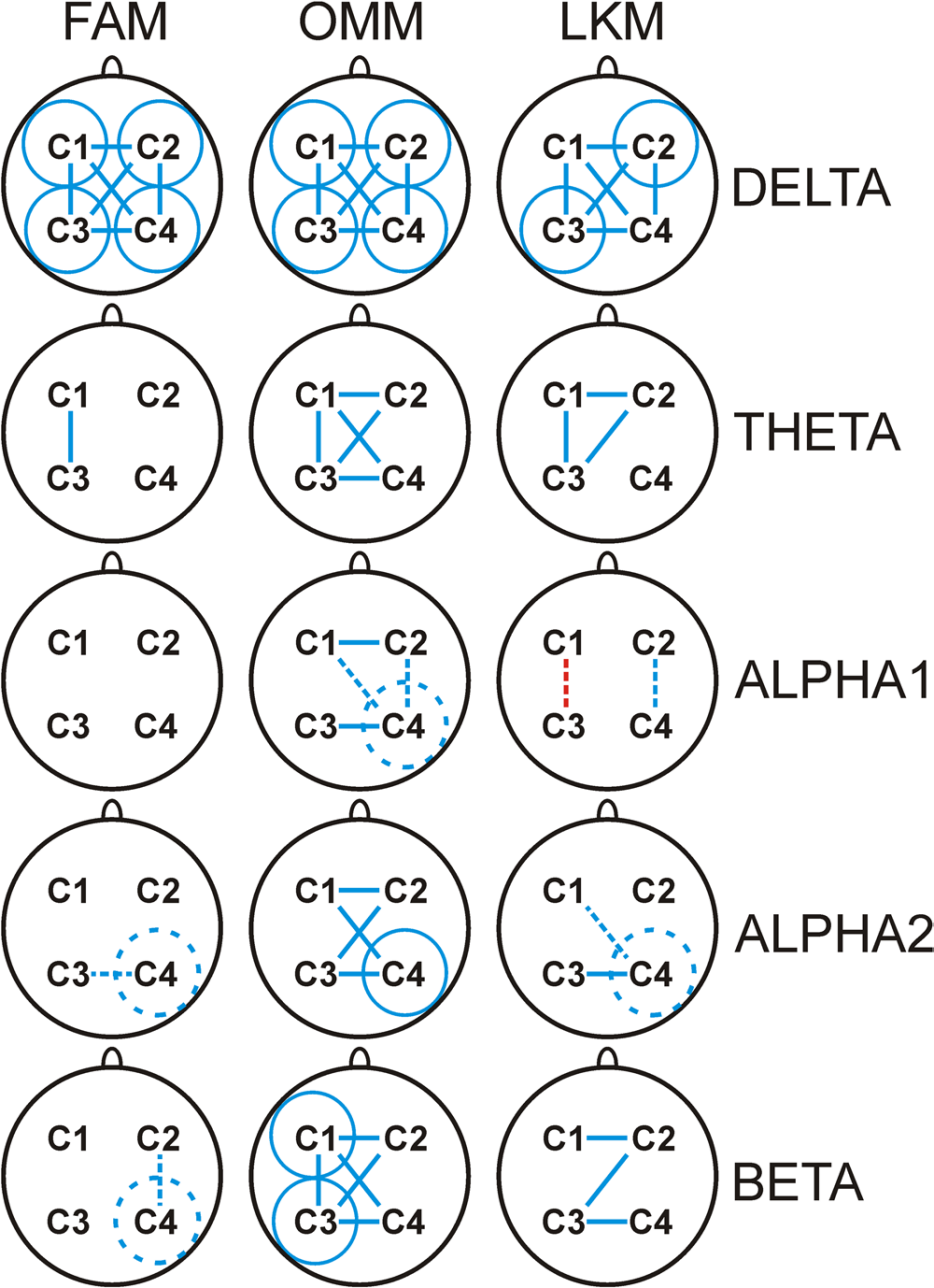
Schematic illustration of statistically significant differences in the re-distribution of strongest and weakest connections (reflected by *ICoh*) between the respective meditation state (FAM, OMM, LKM) and resting state for clusters in delta, theta, slow alpha, fast alpha, and beta frequency ranges. Blue colour indicates meditation-related increase (statistically significant, Bonferroni corrected, p < 3.10^−4^ - continuous line; trend, Bonferroni corrected, 3.10^−4^ < *p* < 3.10^−2^ - dashed line). Circles designate within-cluster (C1, C2, C3, C4) comparisons, lines designate intra- and inter-hemispheric comparisons for C1-C3, C1-C2, C3-C4, etc.

Figure 3 demonstrates statistically significant re-distributions of connectivity in specific meditation conditions. For each cluster, significant increases in the number of pairs with the strongest synchronisation accompanied by decreases in the number of pairs with the weakest synchronisation during meditation are marked in blue, and opposite changes indexing decreases in connectivity strength during meditation are marked in red.

As illustrated in Fig. 3: (1) The synchronisation of delta networks increased for all meditation states for all clusters, with the exception of the C1 and C4 clusters during LKM. This observation points to an overall enhancement of delta connectivity across all three meditation conditions. (2) For all meditation conditions, the synchronisation of theta networks manifested an antero-posterior increase in the left hemisphere (C1-C3), with the additional enhancement of all inter-hemispheric theta connections during OMM, and C2-related inter-hemispheric connections during LKM. These observations reveal a consistent increase in intra-hemispheric theta connectivity in the left hemisphere during all meditation states. Also, the inter-hemispheric connectivity of theta networks increased specifically during OMM condition. (3) No significant re-organisation was detected for slow alpha networks during FAM. However, during OMM, slow alpha synchronisation tended to increase in the right posterior region (C4) and for the intra- and inter-hemispheric connections of C4 and C2 (3.10^−4^ < *p* < 3.10^−2^). Also, during LKM, intra-hemispheric slow alpha synchronisation tended to decrease in the left hemisphere (C1-C3), and to increase in the right hemisphere (C2-C4). (4) The synchronisation of fast alpha networks was significantly re-organised during OMM by being significantly increased in the right posterior region C4, as well as in all inter-hemispheric clusters. Yet, a similar trend for increased connectivity of the right posterior cluster C4 and its inter-hemispheric connections was found for FAM and LKM. (5) The synchronisation of beta networks during OMM manifested a pronounced increase for intra- and inter-hemispheric clusters involving the left hemisphere. During FAM, there was only a trend for increased beta synchronisation in right-hemisphere clusters (C4, C2-C4), and during LKM, inter-hemispheric synchronisation was enhanced.

Together, these observations reveal that all meditation states are characterised by a largely distributed increase of delta connectivity and a prominent increase of theta connectivity in the left hemisphere. OMM is specifically associated with an additional increase of fast frequency synchronisation in alpha and beta frequency bands.

### Condition-specific changes of connectivity during meditation

To assess the effects of meditation states on overall connectivity within and between hemispheres, irrespective of the (re)distribution of strongest and weakest connections, measures of all pairs for each of the 10 clusters were averaged for each subject and condition and were subjected to a repeated measures analysis of variance (ANOVA). For assessment of the effects of types of meditation, difference values were computed by subtracting measures during each meditation (FAM, OMM, LKM) from measures during REST. Following this computation, negative difference values reflect a meditation-related increase, whereas positive difference values reflect a decrease. The first ANOVA design used intra-hemisphere clusters C1, C2, C3, C4, C1-C3, and C2-C4 to assess the effects of meditation on intra-hemispheric connectivity. There were four within-subjects variables: Frequency band (delta, theta, slow alpha, fast alpha, beta), Laterality (Left hemisphere – C1, C3, C1-C3 vs. Right hemisphere – C2, C4, C2-C4), Cluster with 3 levels (anterior – C1, C2 vs. posterior – C3, C4 vs. antero-posterior – C1-C3, C2-C4), and Meditation Condition (FAM, OMM, LKM). The second ANOVA design included the within-subjects factors Frequency band, Cluster (C1-C2, C1-C4, C2-C3, C3-C4) and Meditation Condition (FAM, OMM, LKM) to assess the effects of meditation types on inter-hemispheric connectivity. For within-subjects variables with more than 2 levels, the Greenhouse-Geisser correction was applied to control for sphericity effects. Original *df* and corrected *p* values are reported.

The analysis of the effects of meditation on *ICoh* of intra-hemispheric clusters yielded two significant interactions - Frequency Band x Laterality (F(4/84) = 2.7, p < 0.05) and Frequency Band x Laterality x Meditation Condition (F(8/168) = 2.4, p = 0.05), indicating that types of meditation differentially affected frequency-specific intra-hemispheric connectivity in the left and in the right hemisphere. No significant main or interaction effects were observed for inter-hemispheric synchronisation.

To explore further the effects of meditation states on lateralised intra-hemispheric synchronisation, ANOVAs were conducted for each frequency band. A significant lateral asymmetry was observed for each band, with exception of delta.

**Figure 4:**
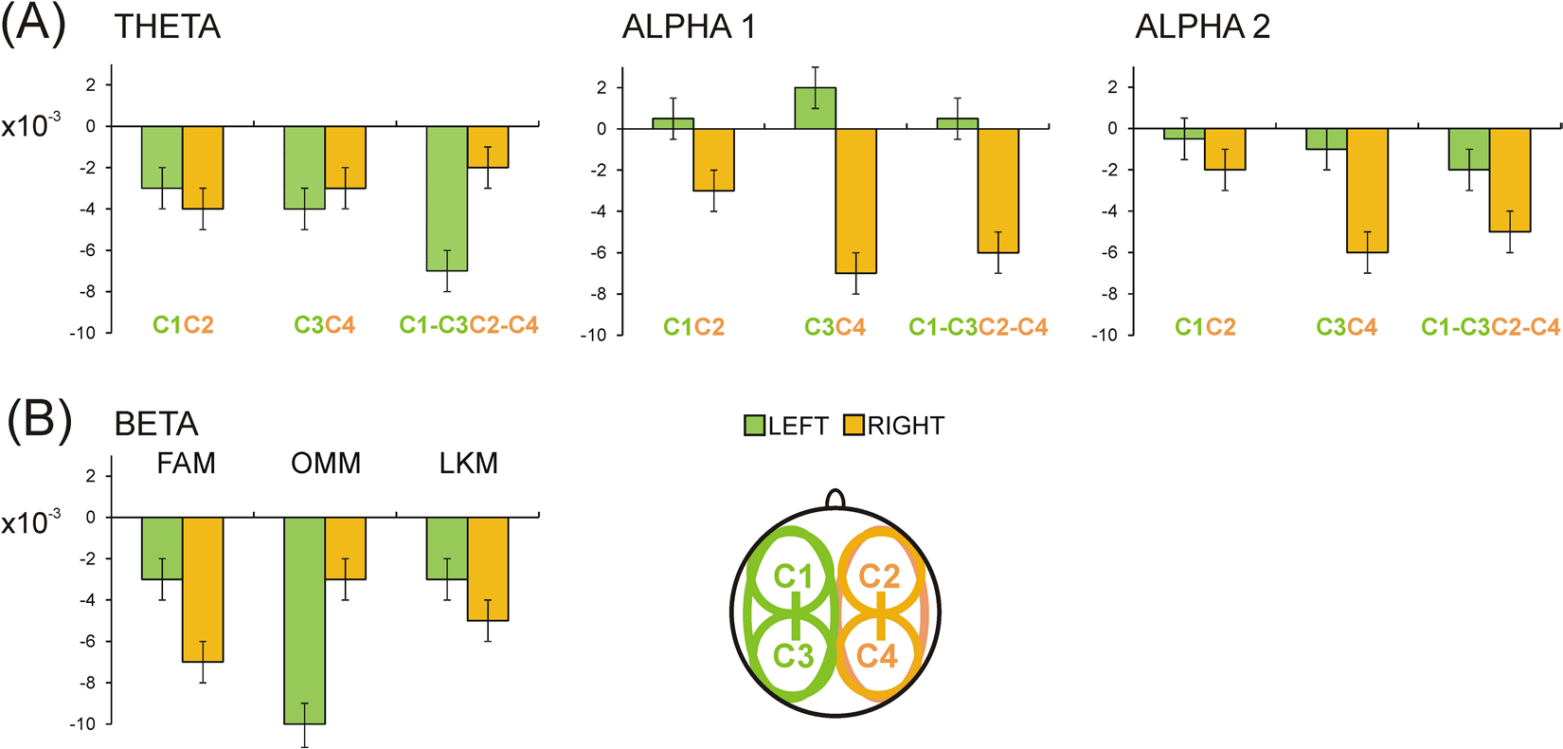
(**A**) Mean values ± standard error of *ICoh* difference between REST and respective meditation conditions (FAM, OMM and LKM) pooled together, for theta, alpha 1 and alpha 2 frequency bands for left-hemisphere clusters (C1, C3, and C1-C3) and right-hemisphere clusters (C2, C4, and C2-C4). Left vs. Right asymmetry common for all meditation conditions is illustrated. (**B**) Mean values ± standard error of *ICoh* difference between REST and respective meditation conditions (FAM, OMM and LKM) for the beta frequency range for left-hemisphere clusters C1, C3, and C1-C3 pooled together, and right-hemisphere clusters C2, C4, and C2-C4 pooled together. Left vs. Right asymmetry differentiating the three meditation conditions is illustrated.

Figure 4A demonstrates that: (1) For all meditation states intra-hemispheric theta synchronisation significantly increased in the left hemisphere (Laterality x Cluster F(2/42) = 3.9, p = 0.03; Laterality effect for C1-C3/C2-C4, F(1/21) = 3.7, p < 0.05; for C1/C2 and C3/C4, F(1/21) <0.3, p > 0.5); (2) In contrast, for all meditation states intra-hemispheric slow and fast alpha synchronisation significantly increased in the right hemisphere (Laterality, F(1/21) > 4.4, p < 0.05), with the strongest enhancement in the right posterior C4 cluster (Laterality x Cluster, F(2/42) > 4.1, p < 0.02). Accordingly, significant Laterality differences were yielded for C3 vs. C4 clusters for both slow (F(1/21) = 8.3, p = 0.009) and fast alpha (F(1/21) = 4.3, p = 0.05) synchronisation; (3) As demonstrated in Fig. 4B, a highly asymmetric lateralisation of intra-hemispheric beta connectivity characterised specific meditation states (Laterality x Condition F(2/42) = 5.1, p = 0.01). During FAM, beta connectivity predominantly increased in the right hemisphere, whereas during OMM – in the left hemisphere, with no reliable left vs. right differences during LKM.

Taken together, these results reveal an overall meditation-unspecific enhancement of intra-hemispheric theta synchronisation in the left hemisphere and of local alpha synchronisation in the right posterior hemisphere. However, lateralised increases in intra-hemispheric beta synchronisation differentiated specific meditation states. In view of the co-occurring lateralised theta and alpha synchronisations it was tested if increased frequency-specific synchronisations in the left and the right hemisphere were associated or independent. Pearson correlational analyses were conducted for each meditation condition to explore if the intra-hemispheric theta increase in the left hemisphere as captured by cluster C1-C3 was linked to the intra-hemispheric/and local posterior slow and fast alpha increase in the right hemisphere as captured by clusters C2-C4 and C4. For FAM and OMM conditions, there were significant moderate correlations between the left-lateralised increase in theta connectivity and right-lateralised increase in slow and fast alpha connectivity (r = 0.4–0.5, p < 0.05), whereas no associations were yielded for LKM. In addition, increased theta synchronisation in the left hemisphere during LKM was not associated with decreased synchronisation of slow alpha in the left hemisphere specifically found during LKM.

### Condition-specific changes of integrating connectivity during meditation

The parameter used for assessing integrating connectivity (*IC*) was inferred by the possibility that some network nodes may act as “hubs” by manifesting strongest connections with all other components of the network, thus taking the position of a “coordinator”^76^. *IC* was computed for each single electrode by averaging *ICoh* values of all pairs linked to that particular electrode. For each electrode the average of connections with all other 49 electrodes was calculated (see ‘Procedure’ in section 4.3.). Thus, the *IC* represents how strongly a localized cortical region represented by a specific electrode is connected with the cortical environment. To compare the effects of different meditation states, difference *IC* values were calculated for each electrode location by subtracting *IC* during meditation from *IC* during rest. Following this computation, negative *IC* differences reflect meditation-related increase, whereas positive *IC* differences reflect a decrease.

Difference *IC* values were subjected to ANOVA with three within-subjects variables Meditation Condition (FAM vs. OMM vs. LKM), Region (F7/8, F3/4, F1/2, FC5/6, FC3/4, FC1/2, C5/6, C3/4, C1/2, CP5/6, CP3/4, CP1/2, P7/8, P5/6, P3/4, P1/2, PO7/8, PO3/4, O1/2) and Laterality (Left – F7, F3, F1, FC5, FC3, FC1, C5, C3, C1, CP5, CP3, CP1, P7, P5, P3, P1, PO7, PO3, O1 vs. Right – F8, F4, F2, FC6, FC4, FC2, C6, C4, C2, CP6, CP4, CP2, P8, P6, P4, P2, PO8, PO4, O2).

**Figure 5:**
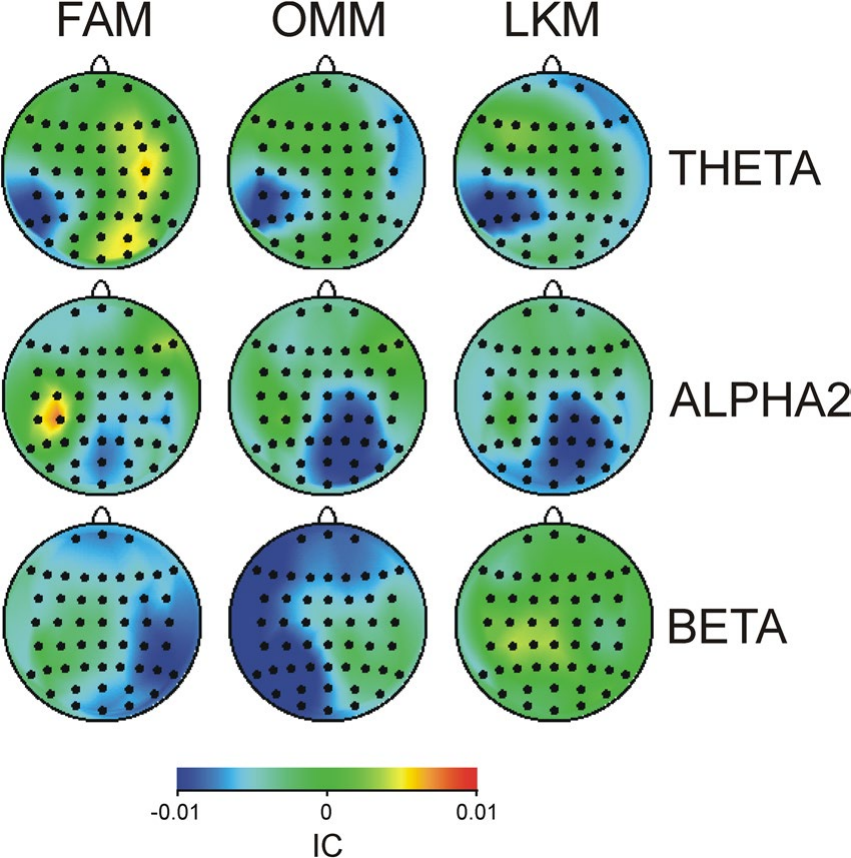
Topography maps of grand average integrated connectivity (*IC*) presented as a difference between REST and the respective meditation condition (FAM, OMM, and LKM) for three frequency ranges (theta, alpha 2, and beta), which showed systematic differences. Blue colour indicates meditation-related increase; red colour indicates meditation-related decrease.

Figure 5 demonstrates difference maps of *IC* for three meditation conditions. No significant effects were yielded in the delta band. In the theta band, *IC* increased for the left posterior (parietal and centro-parietal) regions in each meditation state, as indexed by the significant Region x Laterality interaction (F(19/399) = 2.7, p < 0.05) and a lack of interactions with Meditation Condition (p > 0.8). Testing simple effects revealed that the effect of the Region variable was significant in the left (F(19/399) = 5.3, p = 0.001), but not in right hemisphere (p > 0.6).

*IC* of slow alpha increased at frontal and right centro-parietal regions during FAM and OMM, whereas LKM was characterised by *IC* decrease at left posterior regions (Laterality, (F1/21) = 3.7, p < 0.05). Yet, simple effects of Meditation Condition were only significant in the left hemisphere (F(2/42) = 3.8, p < 0.05). In contrast, Fig. 5 illustrates that all meditation conditions were associated with an increase of *IC* of fast alpha at right parieto-occipital electrodes (Region x Laterality, F(19/399) = 2.8, p < 0.05). Accordingly, no significant interactions with Meditation Condition were yielded (p > 0.3), and the effect of Region was only significant in the right hemisphere (F(19/399) = 3.7, p < 0.05).

Integrating connectivity in the beta range strongly depended on the type of meditation as it increased in the right hemisphere during FAM, in the left hemisphere during OMM, and did not manifest left vs. right hemisphere differences during LKM (Meditation Condition x Laterality, F(2/42) = 3.2, p = 0.05). The lateral asymmetry differences between FAM and OMM were most expressed at posterior (parietal) regions (Meditation Condition x Region x Laterality, F(38/798) = 2.6, p < 0.05). Accordingly, the region-specific effects of Meditation Condition were found for each hemisphere (F(38/798) > 2.3, p < 0.05) – Fig. 5.

Together, these observations indicate that for all meditation conditions integrating connectivity increases in the left parietal regions in theta band, and in the right centro-parietal-occipital regions in the slow and fast alpha bands. Integrating connectivity in the beta band specifically differentiates the types of meditation as it appears specifically lateralised on the right for FAM and on the left for OMM. In addition, only for LKM, we found a pronounced reduction of fast alpha integrating connectivity in the left hemisphere.

### Relationships with meditation expertise

Difference *ICoh* measures of all clusters and difference *IC* measures from five frequency bands in each meditation condition were included in linear and quadratic regression models to test if linear or U-shaped dependencies might exist between meditation-specific changes in synchronisation and Meditation Expertise (life-long duration of meditation practice in hours). None of the models extracted expertise as a significant predictor of synchronisation patterns.

## Discussion

We characterised EEG connectivity patterns during FAM, OMM and LKM in long-term virtuoso meditators in order to investigate (1) whether these meditation types cultivate a generic mental state, (2) or whether these meditation states are profoundly different, despite their apparent commonalities, (3) or whether they share some commonalities while also exhibiting distinct features.

We employed three complementary analytic approaches, which assessed the imaginary part of EEG coherence. This analysis provided converging evidence that the three meditation conditions share a highly consistent connectivity pattern as compared to resting state. As also summarised in Table 1, this pattern is characterised by three features: (a) a largely distributed increase of delta connectivity; (b) a prominent increase of intra-hemispheric theta connectivity within the left hemisphere, with a local integrating focus in the left posterior hemisphere; and (c) a prominent increase of intra-hemispheric slow and fast alpha synchronisation within the right hemisphere, with a local integrating focus in the right posterior regions. These results suggest that the three meditation states share a potentially unique underlying brain state that is clearly distinguished from rest: brain networks in different frequency bands become strongly connected, encompassing in an asymmetric manner the left and right hemisphere, independently of the type of meditation (FAM, OMM, or LKM).

**Table 1:** Summary of the main findings, indicating the increased connectivity patterns for each meditation type, compared to the REST condition.

	FAM	OMM	LKM
**DELTA**			
Broadly distributed increase	✓	✓	✓
**THETA**			
Left-hemispheric increase with posterior integrating focus	✓	✓	✓
**FAST ALPHA**			
Right-hemispheric increase with parieto-occipital cluster	✓	✓	✓
**BETA**			
Left-hemispheric increase		✓	
Right-hemispheric increase	✓		
**THETA – FAST ALPHA**			
Inter-hemispheric correlation between left-hemispheric THETA and right-hemispheric FAST ALPHA	✓	✓	

The results, furthermore, indicate that in addition to the shared features of neural activity, each meditation state expresses specific synchronisation patterns. Notably, these condition-specific patterns (a) manifest in the higher frequency beta band, and (b) exhibit hemispheric lateralisation (see Table 1). These observations provide evidence for the existence of a lateralised neural substrate that specifically supports mental processes unique for each meditation type.

Thus, the present study suggests the coexistence of an underlying large-scale *matrix* of coherent oscillations shared by different forms of meditation, and of dynamic *cores* related to specific forms of meditation, in terms of differential synchronisation patterns in the brain.

Specifically, we found a shared involvement of **delta** coherent oscillations in all forms of meditations, in contrast to the non-meditative rest condition. According to Lutz *et al*.^62^, “… the brain goes through a succession of large-scale brain states, with each state becoming the source of top-down influences for the subsequent state. We predict that these large-scale integrative mechanisms participate in the regulatory influence of these meditation states.” (p. 5). In line with this perspective, the observed broadly distributed increase in delta connectivity in all meditation conditions points to the delta band as a main frequency associated with large-scale integrative mechanisms subserving these meditation states^62^. Given that meditation practice generally enhances vigilance and meta-cognition in the present moment, large-scale delta coherence also might relate to a “global neuronal workspace” or “dynamic core” processes in the brain^58,59,61,67,68,77–79^ and provide a neurodynamic substrate for enhanced conscious access in different forms of meditation^43,61,62^.

The present results further reveal that the functional connectivity or synchronisation patterns in the **theta** band was increased in the left hemisphere in all forms of meditation. This result expands evidence from previous studies^15,16^ that used fMRI and MEG, respectively, and highlighted the involvement of areas in the left hemisphere during FAM and OMM. Similarly, the meta-analysis of functional neuroimaging studies by Fox *et al*.^49^ identified a prominent cluster of left-hemispheric activation across meditation types. More specifically, Hölzel *et al*.^80^ reported increased grey matter in the left hippocampus of mindfulness meditators. Another structural neuroimaging study^81^ reported structural differences of long-term meditation practitioners in the superior longitudinal fasciculus, a major association fibre tract that connects frontal, occipital, parietal and temporal lobes, a difference that was more pronounced in the left hemisphere. Another recent study^82^ of long-term meditators demonstrated a leftward grey matter asymmetry in the superior parietal lobe, a key region involved in the control of attention^70^ and of executive functions^56^. These structural neuroimaging findings suggest increased leftward grey matter asymmetries in the set of associative cortical regions subserved by the superior longitudinal fasciculus. Our results evidence more dynamic aspects, demonstrating the involvement of coherent theta oscillations in the left hemisphere as a putative functional correlate of such leftward structural asymmetries. The presence of this effect in all meditation conditions implies that along with delta, also the theta frequency can be related to an underlying matrix of large-scale coherent oscillations supporting intensified processes of consciousness within the global neuronal workspace model^78^. The left-hemisphere localisation and the presence of a localised centre of increased connectivity in the left posterior regions, however, imply a more specialised role for theta in meta-cognition rather than generally providing for a facilitated access to enhanced consciousness. In the experienced meditators studied here, this may reflect neuroplasticity either in the system for semantic processing in the left hemisphere^73^, a reorganisation in the theta-related systems of attentional control^71^, or a specific functional cluster (dynamic core) of higher-order consciousness^82,83^.

In addition, the coherence patterns in the **fast alpha range** reflect increased connectivity also in the right hemisphere, including a common connectivity cluster in the right parieto-occipital regions in all forms of meditation. Such common involvement might be related to a brain network in the alpha band for “inhibition and timing” related to gating and selective enhancement of sensory inputs^84^ or to pulsed-inhibition of ongoing cortical processing^85,86^. Fast alpha coherence in the right parieto-occipital cortex may thus reflect a facilitated ‘reset’ mechanism in the discrete transition from one perceptual dynamic state to another^61,62,77,79^, enabling an enhanced top-down regulation of sensory inputs in meditation. The right-hemispheric focus of increased connectivity in the right parieto-occipital region may reflect the presence of a unique system in the right human brain for pulsed attentional gating of sensory information, which operates in the alpha frequency band to subserve the still not well understood link between attention and consciousness. Alternatively, there may be a neuroplastic frequency shift to higher frequencies in trivial right-hemisphere attentional networks controlling arousal, novelty and distraction that typically operate in the theta frequency band^70–72^.

Notably, a positive correlation between theta connectivity in the left hemisphere and alpha connectivity in the posterior right hemisphere was only found for FAM and OMM. This correlation suggests interdependence and coordination between brain signals in the two hemispheres during meditation and emphasises the possibility that groups of distributed but coordinated functional clusters (dynamic cores) may underlie attentional control in enhanced higher-order consciousness and meta-cognition^59,83^.

According to the present results, a lateralisation of coherence in the **beta** band distinguished the three meditation conditions most reliably. Enhanced beta coherence was lateralised to the left hemisphere in OMM, and to the right hemisphere in FAM, whereas no inter-hemispheric asymmetry was observed in LKM. Although beta oscillations have been linked with multiple functions^87^, they are mostly associated with endogenous, top-down-controlled processing^69,88^, as well as with conscious access processes^68,74,75^ by supporting long-range re-entrant interactions between cortical areas and enhanced “communication through coherence”^88,89^. The prominent difference in the involvement of the two hemispheres across meditation conditions implies a specific role for increased beta connectivity in conscious access control.

Notably, the most prominent difference in the lateralisation of increased beta connectivity was between FAM and OMM. Both FAM and OMM can be operationalised as regulating attention and meta-cognition but with different apertures, FAM with a narrow attentional aperture and OMM with the widest possible aperture^18,31,38^. We may, therefore, speculate that increased beta connectivity in the right hemisphere reflects highly focused attention on an object, with a concurrent gating of sensory inputs, characterising FAM. In contrast, increased beta connectivity in left hemisphere corresponds to wide attention to all present moment experiences, characterising OMM^17,29,38,62^. Hence, lateralised beta connectivity may reflect either the type of attentional process of selection (narrow/focused vs. wide/monitoring) or the amount of selected information (small vs. large). Relatedly, lateralised beta connectivity may be associated with amplified awareness of selected contents in the fields of experience and mental state factors^68,74,75^. Direct associations with mere activation of attentional systems are not plausible because (a) the meditators studied here were all highly experienced and supposedly practiced effortless meditation, and (b) no correlations with meditation expertise were revealed for beta band connectivity. In line with these interpretations, in LKM, no lateralised patterns of enhanced beta connectivity were observed, with only interhemispheric beta coherence being enhanced.

Taken together, our findings highlight the importance of studying large-scale coherent oscillations and coordinated interactions of brain networks for increasing the understanding of the neural bases of meditation. Here, for the first time, by using *ICoh* as a measure of neural coupling, we demonstrated its usefulness in revealing commonalities and differences of three meditation states. Our results, thus, provide new evidence that focused attention, open monitoring and loving kindness meditation share neural mechanisms possibly supporting heightened states of attention and of meta-cognitive processes, or generally enhanced conscious access.

The patterns of coherent oscillatory activity that were meditation-specific confirm that different meditation instructions and associated phenomenological experiences, are indeed reflected in neural activity. We interpret these differences in terms of narrow vs wide apertures of attention or as a small vs large amount of attended information, for FAM and OMM, respectively.

The study, thus, brings us a step closer to understanding the specific neural effects of these meditation types. It highlights that engaging with any of them is likely to lead to generally enhanced neural coupling, engaging differentially theta and alpha networks in the left and right hemispheres. In addition, the different types of meditation may also lead to meditation-specific lateralised changes in fast-frequency beta networks.

Subsequent studies can build on these findings to gain a more fine-grained understanding of dynamic coupling during meditation, including the promising investigation of oscillatory frequency coupling^66^, and directed connectivity and large-scale neuro-computational modelling of neural integration and conscious access processes^60,78,90^.

## Materials and Methods

### Participants

We studied 22 healthy right-handed volunteers (mean age = 44.2 years, age range 26-70 years, 4 females) who did not report any history of movement disorders or neurological diseases. The participants were monks, nuns and novice practitioners residing at Amaravati Buddhist Monastery, in Southern England, and at Santacittarama Monastery, in Central Italy. Practices at both monasteries are aligned with the Thai Forest Theravada Buddhist tradition which is now established, widely acknowledged and influential in the West. Participants practiced FAM (*Śamatha*), OMM (*Vipassanā*) and LKM (*Metta*) meditation forms in a balanced way in this tradition, often in integrated sessions, including silent meditation retreats (3 months per year). Meditation expertise is measured in hours taking into account both practice in the monastic tradition and practice before monastic life. In this tradition, the monks, nuns and novice practitioners typically practice two hours per day with the monastery community, with a regular intensification of practice during retreats (with several meditation sittings during the 3-month Winter retreat). As suggested by the abbots of the monasteries, we estimated an average of 100 hours of practice per month during monastic life, with a balance of FAM, OMM and LKM facets of meditation. The lifetime duration of meditation practice of the participants was estimated as a mean value = 19358 hours (SE = 3164), range 900-50600 hours.

The study had prior approval by the dedicated Research Ethics Committee at Sapienza University of Rome, Italy. All participants gave informed consent before participation according to the Declaration of Helsinki.

### Experimental design

Participants had to perform a non-meditative rest condition and three meditation conditions: FAM, OMM and LKM. The switching between conditions was cued by voice. The instructions for the four conditions, which were written together with the abbot of Amaravati Monastery, the internationally recognized teacher Ajahn Amaro, were as follows:

*Rest*: “Rest in a non-meditative relaxed state, without falling in sleep, while allowing any spontaneous thoughts and feelings to arise and unfold in the field of experience”.

*Focused attention (Śamatha) meditation (FAM): “*Sustain the focus of attention on breath sensations, such as at the nostrils, noticing readily and with acceptance any arising distraction, such as on thoughts or stimuli, and in case of detected distraction, return readily and gently to focus attention on the breath sensations”.

*Open monitoring (awareness) meditation (OMM):* “With an open receptive awareness, observe the contents of experience as they arise, change and fade from moment to moment, without restrictions or judgments – such contents including breath and body sensations, sensations arising from contact with external stimuli, feelings and thoughts”.

*Loving kindness (Metta) meditation (LKM):* “Generate and sustain *metta*, acceptance and friendliness towards yourself and the experience in the present moment, as well as towards any being, in any state or condition”.

### Procedure, EEG recordings and pre-processing

EEG was recorded with eyes closed in blocks of duration of approximately 2.5-3 minutes each while the above mentioned conditions were repeated two times. In such a way, four blocks were repeated twice in the following order: Rest, FAM, OMM, LKM. Thus, there were approximately 5-6 minutes (2 blocks x 2.5-3 minutes) of total recording for each condition. EEG was acquired by a mobile wireless system produced by Cognionics (https://www.cognionics.net/mobile-128) using an electrode cap with 64 active Ag/AgCl electrodes located in accordance with the extended international 10/20 system and referenced to linked mastoids. Electrode impedances were kept below 10 kOhm and EEG signals were collected at a sampling rate of 500 Hz (reduced off-line to 250 Hz for data analysis). The experiment was conducted at the two monasteries in a quiet, dark room suitable for meditation and recording EEG. Participants were tested one at a time.

Analyses were performed by means of Brain Vision Analyzer 2.2 (Brain Products GmbH, Germany) and by custom software developed on Matlab R2013b (The MathWorks Inc.). EEG traces were visually inspected to reject epochs with noise or non-physiological artifacts. Bad channels were interpolated according to Hjorth^91^. For control of ocular artifacts, vertical and horizontal electro-oculogram (EOG) was also recorded and all EEG traces were EOG corrected by means of independent component analysis (ICA)^92^.

To improve spatial resolution and reduce the volume conduction between electrodes, current source density was additionally applied with the parameters: order of splines = 10, maximal degree of Legendre polynomials = 4, lambda = 1E^-7^. Edge electrodes were excluded from all analyses, so that the number of channels was finally reduced to 50. All analyses were carried out with CSD transformed data from 50 electrodes.

### Transformation of EEG signals to frequency domain

EEG recordings from all conditions were segmented in equal-sized epochs of 4.096-s duration with 1.024 s overlap. The average number of epochs for each condition/participant was 70 (±20). After Hanning window with a duration of 20% from the total epoch length was applied to all epochs, the fast Fourier transform was computed, yielding the representation of complex values (real and imaginary parts) with a frequency resolution of 0.244 Hz (1/4.096 s).

### Imaginary part of coherence

Coherence function is a measure of the linear relationship between two signals at a specific frequency. In practice, the magnitude of the coherency function is generally used as a measure of coherence. Coherence function *C*_*xy*_*(f)* is defined as the normalized cross-spectrum:

$${C}_{xy}(f)=\frac{\sum Sxy(f)}{\sqrt{\sum Sxx(f)\sum Syy(f)}},$$

where 𝑆𝑥𝑦 is the cross-spectral density between two signals, and 𝑆𝑥𝑥 and 𝑆𝑦𝑦 are the autospectral densities for signals 𝑥 and 𝑦, respectively. Coherence is defined as the magnitude of coherence function:

$$Co{h}_{xy}(f)=|{C}_{xy}(f)|=|\frac{\sum Sxy(f)}{\sum Sxx(f)\sum Syy(f)}|.$$

The estimated coherence for a given frequency ranges between 0 and 1, with a value of 0 indicating that the two signals are perfectly uncorrelated, and a value of 1 indicating the perfect correlation.

Instead of estimating the magnitude of the coherence function, Nolte *et al*.^44^ proposed using only the imaginary part of the coherency to investigate brain interactions. This is because the imaginary part of the coherency excludes coherent sources with zero phase lag and therefore reduces the effect of field spread due to volume conduction. The imaginary part of coherency is defined as

$$ICo{h}_{xy}(f)=Im({C}_{xy}(f))=Im\left(\frac{\sum Sxy(f)}{\sum Sxx(f)\sum Syy(f)}\right),$$

where *Im* denotes the imaginary part. The imaginary part of coherency for a given frequency varies between -1 and 1. If the value is positive, signals 𝑥 and 𝑦 are interacting and 𝑦 is earlier than 𝑥, indicating that information is flowing from 𝑦 to 𝑥.

### Connectivity analysis of EEG signals

*ICoh* is a refined measure of connectivity, because it is not contaminated by volume conduction and by the contribution of common sub-cortical sources. This is so because, in contrast to the coherence measure, *ICoh* captures only stable phase differences between single electrodes and disregards fully coherent signals, which may be produced by either volume conduction or by a common synchronising source. Thus, *ICoh* reliably reflects the true synchronisation between cortical regions. Volume conductance was additionally controlled by using CSD (applied previously to EEG).

The imaginary part of coherency is only sensitive to functional coupling between two signals which are time-lagged to each other. Therefore, as far as volume conduction does not cause a time-lag, this measure is less sensitive to common sources effects^44^.

*ICoh* was obtained for all 1225 combinations (for 50 electrodes) for each condition and each participant. In this study, we investigated the absolute value of *ICoh*^65^. Additionally, before submitting it to statistical analysis, *ICoh* was three-point smoothed and, in order to normalize distribution, Fisher z-transformation was applied. Fisher z-transformation is given by the equation: $z=\frac{1}{2}ln\left(\frac{1+{\rm{r}}}{1-{\rm{r}}}\right)=arctanh(r)$, where *ln* is the natural logarithm, *arctanh* is the inverse hyperbolic tangent function, and *r* is the actual value.

Analyses were done in two directions: selected electrode pairs and all electrodes pooled together. In order to perform data reduction and present more generalized data presentation, data pooling was performed in the following way: all electrode pairs containing one and same electrode were averaged and recorded with the name of this electrode. In such a way, information about each electrode and its connections to all other electrodes was obtained, underlining the strength of connectivity with other electrodes.
